# Enhancement in colonization of bovine spermatogonial stem cells following addition of knock-out serum replacement to culture medium

**Published:** 2016-12-15

**Authors:** Reza Youssefi, Parviz Tajik, Mansoureh Movahedin, Vahid Akbarinejad

**Affiliations:** 1 Department of Theriogenology, Faculty of Veterinary Medicine, University of Tehran, Tehran, Iran; 2 Department of Anatomical Sciences, Faculty of Medical Sciences, Tarbiat Modares University, Tehran, Iran; 3 Young Researchers and Elites Club, Roudehen Branch, Islamic Azad University, Roudehen, Iran

**Keywords:** Bovine, Colonization, Fetal bovine serum, Knock-out serum replacement, Spermatogonial stem cell

## Abstract

Enrichment of cell suspension with germ cells prior to injection into recipient seminiferous tubules is of importance in spermatogonial stem cells (SSCs) transplantation. Knock-out serum replacement (KSR) has been reported to enhance the proliferation of murine SSCs and human embryonic stem cells. The aim of the present study was to investigate the effect of KSR versus fetal bovine serum (FBS) and their interaction on colonization of bovine SSCs in vitro. When FBS (10%) was replaced with KSR (10%), a significant increase in the colonization of SSCs and the expression of Thy1, as marker for enrichment of SSCs, was observed. It was revealed that the lesser proliferative effect of FBS as well as the greater proliferative impact of KSR on SSCs colonization were not irreversible as cells having been cultured with FBS (10%) for three days with low colonization showed high rate of colonization in response to KSR (10%) and cells having been cultured with KSR (10%) with high colonization experienced low rate of colonization in response to FBS (10%). Further, it was shown that FBS did not contain factors inhibiting SSCs colonization and it simply lacked factors essential for SSCs proliferation because the combination of FBS (5%) and KSR (5%) resulted in even greater rate of colonization than did KSR (10%). In conclusion, the present study showed that addition of KSR to culture medium would significantly increase SSCs proliferation.

## Introduction

Spermatogonial stem cells (SSCs) are uniquely capable of transferring genetic information to the next generation. As a result, transplantation of SSCs has opened a novel perspective in biotechnology for generation of transgenic animals.^1^ SSCs transplantation is well-established in murine,^2^^,^^3^ and its efficiency has been enhanced over the last decades.^4^ In bovine, in spite of some trials for SSC transplantation,^5^^-^^7^ highly successful transplantation of bovine SSCs― leading to long term regeneration of spermatogenesis in the recipients― remained to be comprehensively established.^1^ One of the cornerstones for enhancing the efficiency of SSCs transplantation is the enrichment of the donor-derived testicular cell suspension prior to injection into the recipient seminiferous tubules due to the rarity of SSCs in mammalian testes.^1^

In vitro culture is one of the methods for enrichment of donor cell population before transplantation.^1^^,^^8^^-^^10^ In this context, various culture conditions could influence the expansion of SSCs, for example, Aponte et al. reported that supplementation of SSCs culture with stem cell medium as compared with minimal essential medium would result in greater proliferation of SSCs.^10^ Knock-out serum replace-ment (KSR), which was originally developed for culture of embryonic stem cells,^11^ has been successfully used for testis organ culture^12^ and serum- and feeder-free culture of spermatogonial^11^ as well as embryonic stem cells.^13^ Moreover, Garcia-Gonzalo and Izpisúa Belmonte found that KSR stimulate the proliferation and self-renewal of human embryonic stem cells.^13^

The present study was conducted to investigate the effect of KSR as compared with fetal bovine serum (FBS) as well as the interaction between KSR and FBS on bovine SSCs colonization, which results from the interactions between SSCs and Sertoli cells during invitro culture.^8^^-^^10^

## Materials and Methods

Animals and testicular biopsy. Animal Ethics Committee at University of Tehran approved the present study in terms of animal welfare and ethics. To obtain testicular tissue, Holstein calves (n = 15; five claves were used for each experiment), aged 3 to 5 months were subjected to testicular biopsy as previously described.^14^ In brief, testicular biopsy was performed under sedation with 0.2 mg kg^-1 ^xylazine (Alfasan, Woerden, Holland) and local anesthesia with lidocaine (Aburaihan Pharma Co., Tehran, Iran). Following incision, the testicular tissue was obtained and placed into a15 mL tube containing Dulbecco' minimal essential medium (DMEM; Gibco, California, USA) with 10% fetal bovine serum (FBS; Sigma, Missouri, USA) and antibiotics (100 IU mL^-1^ penicillin and 100 μg mL^-1^ streptomycin; Gibco). The specimen was subsequently transferred on ice to the laboratory within 2 hr.

Experiment 1. In FBS and KSR groups, cells were cultured in wells containing DMEM with 10% FBS and 10% KSR, respectively, for 12 days. The number of colonies was evaluated on days 3, 6, 9 and 12. The gene expression of Thy1 was assessed on day 9, as in the experiments associated with colonization we observed the highest number of colonies on day 9.

Experiment 2. In FBS-FBS group, cells were cultured in wells containing DMEM plus 10% FBS for 12 days. In FBS-KSR group, cells were cultured in wells containing DMEM plus 10% FBS for the first three days, and in wells containing DMEM plus 10% KSR thereafter until day 12 of culture. In KSR-KSR group, cells were cultured in wells containing DMEM plus 10% KSR for 12 days. In KSR-FBS group, cells were cultured in wells containing DMEM plus 10% KSR for the first three days, and in wells containing DMEM plus 10% FBS thereafter until day 12 of culture. The number of colonies was evaluated on days 3, 6, 9 and 12.

Experiment 3. In FBS, FBS+KSR and KSR groups, cells were cultured in wells containing DMEM plus 10% FBS, 5% FBS + 5% KSR and 10% KSR, respectively, for 12 days. The number of colonies was evaluated on days 3, 6, 9 and 12. The gene expression of Thy1 was assessed on day 9.

Cell isolation. Cell isolation was implemented using a two-step enzymatic isolation procedure, as previously described.^14^ In brief, the testicular tissue was washed three times in DMEM containing antibiotics and was minced into small pieces by a sterile scissors. The minced testicular tissue was incubated in DMEM containing 1 mg mL^-1^ collagenase (Sigma), 1 mg mL^-1^ hyaluronidase (Sigma), 1 mg mL^-1^ trypsin (Sigma) and 5 µg mL^-1^ DNase (Fermentas, St. Leon-Rot, Germany) at 37 ˚C in a shaker incubator with 80 cycles min^-1^ for 60 min. Digested testicular tissue was washed three times with DMEM and the supernatant was disposed after each washing, resulting in isolation of seminiferous tubules. During the second step of enzymatic digestion, the seminiferous tubules were incubated at 37 ˚C in DMEM containing 1 mg mL^-1^ collagenase, 1 mg mL^-1^ hyaluronidase and 5 µg mL^-1^ DNase until disintegration of the seminiferous tubules and separation of the constituent cells. Individual cells were isolated from the remaining tubule fragments by centrifugation at 30 g for 2 min. Following filtration through 77 and 55 mm nylon filters, the cells were pelleted. The pellet was re-suspended in DMEM containing antibiotics.

Cell culture. In the experiments related to assessment of gene expression and colonization, 6 and 24-well plates were used, respectively. In the experiments associated with evaluation of gene expression and colonization, cells were seeded at concentrations of 1 × 10^6^ and 3 × 10^5^ cells per well. The plates were incubated at 37 ˚C in a humidified atmosphere with 5% CO_2_. The medium was replaced with fresh one every three days.

Evaluation of the colonization. Five cell populations from different calves were used in each experiment for evaluation of the effect of FBS versus KSR or their interaction on colonization of testicular germ cells. The number of colonies was determined using an inverted microscope (Model IX71; Olympus, Tokyo, Japan).

RNA isolation and quantitative real-time PCR. Following trypsinization of the cultured cells (n = 3 cell populations from different calves), the isolated cells were subjected tototal RNA extraction using Trizol reagent (Fermentas). The extracted RNA was treated with DNase (Fermentas) to eliminate DNA contamination. The concentration of RNA was determined by UV spectro-photometry (Eppendorff, Hamburg, Germany). The cDNAs were synthesized from 500 ng of RNA by oligo (dT) primers using RevertAid™ First Strand cDNA Synthesis Kit (Fermentas). Primer sequences were as follows: 5´-TCGCCCGAGTCCACACAG-3´, 5´-ACCTCAACCCGCTCCCAAG-3´ (β-actin), and 5´-TTCATCTCCTTGTGACGGGTT-3´, 5´-GCAGAGGTGAGGGAATGGC-3´ (Thy1). The PCRs were performed using Master Mix and SYBR Green I (Fermentas) in an Applied Biosystems, StepOne™ thermal cycler (Applied Biosystems, California USA). The PCR program started with an initial melting cycle for 5 min at 95 ˚C to activate the polymerase, followed by 40 cycles of melting (30 sec at 95 ˚C), annealing (30 sec at 58 ˚C) and extension (30 sec at 72 ˚C). The quality of the PCR reactions was confirmed by melting curve analyses. For each sample, the reference gene (β-actin) and Thy1 gene were amplified in the same run. The Thy1 gene was normalized to the reference gene. The mean Thy1 gene threshold cycle (Ct) and mean exogenous control (β-actin) Ct for each sample were calculated from duplicate wells. The threshold cycle (Ct) of the control was subtracted from the Ct of Thy1 gene, resulting in ∆Ct. In each experiment, the Ct of time-point 0 sample was considered as calibrator. Subsequently, the ∆Ct of sample was then subtracted from the ∆Ct of calibrator, resulting in the ∆∆Ct, which was used for calculation of the relative amounts of Thy1 gene expression for each sample.^15^

Statistical analysis. Initially, datasets were tested for normal distribution using Kolmogorov-Smirnov test (uni-variate procedure). Data associated with colonization in experiments 2 and were log (base 10) transformed prior to analysis because of the lack of normal distribution. Data associated with the number of colonies and gene expression were analyzed using GLM procedure. LSMEANS statement was used to perform multiple comparisons. All analyses were conducted in SAS (version 9.2; SAS Institute, Cary, USA). Data are presented as mean ± SD. Differences with p < 0.05 were considered significant.

## Results

**Fig. 1:**
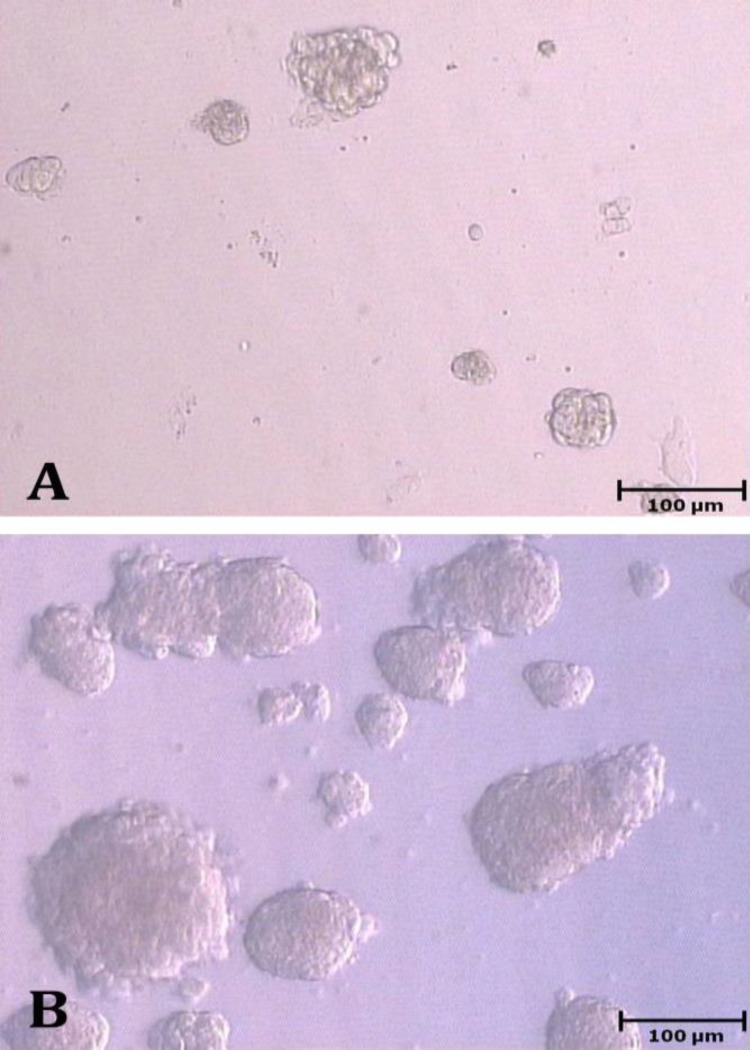
Colonization of spermatogonial stem cells in fetal bovine serum** (A)** and knock-out serum replacement groups on day 9 of culture** (B**

**Fig. 2:**
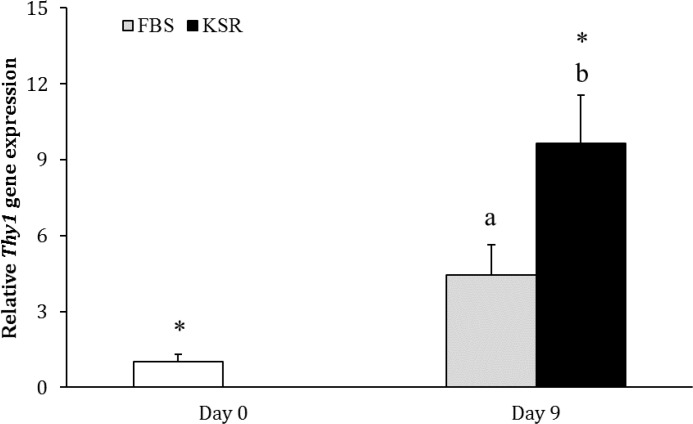
Relative gene expression of *Thy1* in FBS and KSR groups (n = 3) on days 0 and 9. Asterisks indicate significant difference within groups between days 0 and 9 (*p *< 0.05). Different letters indicate significant difference between two experimental groups on day 9 (*p *< 0.05

 **Experiment 1. **The number of colonies did not significantly change during culture in FBS group (*p *> 0.05). In KSR group, the number of colonies constantly increased from day 3 to 9 (*p *< 0.05; Fig. 1), but it decreased from day 9 to 12 (*p* < 0.05; Table 1). The number of colonies did not differ between two groups on day 3; however, it was higher in KSR than FBS group on days 6, 9 and 12 (*p *< 0.05; Table 1). The expression of *Thy1* in FBS group did not change after culture (*p* > 0.05), but it increased from day 0 to 9 in KSR group (9.66 ± 1.90 fold; *p* < 0.05). The gene expression of *Thy1* was higher in KSR than FBS group on day 9 (*p* < 0.05; Fig. 2).

**Table 1:** Number of colonies in fetal bovine serum (FBS) and knock-out serum replacement (KSR) groups on days 3, 6, 9 and 12 of culture. Data are presented as mean ± SD

**Group**	**Days of culture**			
	**3**	**6**	**9**	**12**
**FBS**	0.6 ± 0.5	8.8 ± 4.9 A	17.2 ± 4.4 A	18.8 ± 4.4 A
**KSR**	65.0 ± 10.6 a	142.8 ± 25.2 bB	326.8 ± 65.4 cB	237.2 ± 74.2 dB

abcd different lowercase superscripts within rows indicate significant differences (*p* < 0.05).

AB different uppercase superscripts within columns indicate significant differences (*p* < 0.05).

**Experiment 2. **In FBS-FBS group, the number of colonies did not significantly change during culture (*p *> 0.05). In FBS-KSR and KSR-KSR groups, the number of colonies increased constantly from day 3 to 9 (*p *< 0.05). Afterwards, the number of colonies remained unchanged in FBS-KSR group (*p *> 0.05), whereas it decreased in KSR-KSR group (*p *< 0.05). In KSR-FBS group, the number of colonies did not differ among days 3, 6 and 9, and among days 6, 9 and 12 (*p *> 0.05), but it was lower on day 12 than day 3 (*p* < 0.05). On day 3, the number of colonies in KSR-KSR and KSR-FBS groups was higher than that in FBS-FBS and FBS-KSR groups (*p *< 0.05), but there was no difference between KSR-KSR and KSR-FBS groups, and between FBS-FBS and FBS-KSR groups (*p *> 0.05). On days 6 and 9, the number of colonies in FBS-KSR and KSR-KSR groups was greater than that in FBS-FBS and KSR-FBS groups (*p *< 0.05), also, it was higher in KSR-KSR than FBS-KSR group (*p *< 0.05). But the number of colonies was not different between FBS-FBS and KSR-FBS groups on days 6 and 9 (*p *> 0.05). On day 12, the number of colonies in FBS-KSR and KSR-KSR groups was greater than that in FBS-FBS and KSR-FBS groups (*p* < 0.05), but there was no difference between FBS-FBS and KSR-FBS groups (*p *> 0.05), and between FBS-KSR and KSR-KSR groups (*p *> 0.05; Table 2).

**Experiment 3. **In FBS group, the number of colonies was not significantly different among days 3, 6, 9 and 12 (*p *> 0.05). In FBS+KSR group, the number of colonies constantly increased from day 3 to 9 (*p *< 0.05); however, it decreased from day 9 to 12 (*p* < 0.05). In KSR group, the number of colonies increased from day 3 to 9 (*p *< 0.05), but it remained unchanged thereafter (*p *> 0.05; Table 3). On day 3, the number of colonies was higher in FBS+KSR and KSR groups compared with FBS group (*p *< 0.05), but it was not different between FBS+KSR and KSR groups (*p* > 0.05). On days 6, 9 and 12, the number of colonies was greater in FBS+KSR group than in FBS and KSR groups (*p *< 0.05); additionally, it was higher in KSR compared with FBS group (*p *< 0.05; Table 3).

**Table 2:** Number of colonies in fetal bovine serum (FBS)-FBS, FBS-knock-out serum replacement (KSR), KSR-KSR and KSR-FBS groups on days 3, 6, 9 and 12 of culture. Data are presented as mean ± SD

**Group**	**Days of culture**			
	**3**	**6**	**9**	**12**
**FBS-FBS**	0.6 ± 0.5 A	9.2 ± 3.9 A	19.0 ± 3.1 A	19.6 ± 3.2 A
**FBS-KSR**	0.4 ± 0.5 aA	91.8 ± 7.1 bB	239.0 ± 19.6 cB	220.2 ± 15.5 cB
**KSR-KSR**	60.6 ± 16.7 aB	158.6 ± 20.8 bC	352.8 ± 53.0 cC	257.8 ± 34.6 dB
**KSR-FBS**	63.2 ± 13.3 aB	37.8 ± 13.4 abA	28.6 ± 13.8 abA	20.8 ± 5.0 bA

abcd different lowercase superscripts within rows indicate significant differences (*p* < 0.05).

ABC different uppercase superscripts within columns indicate significant differences (*p* < 0.05).

**Fig. 3:**
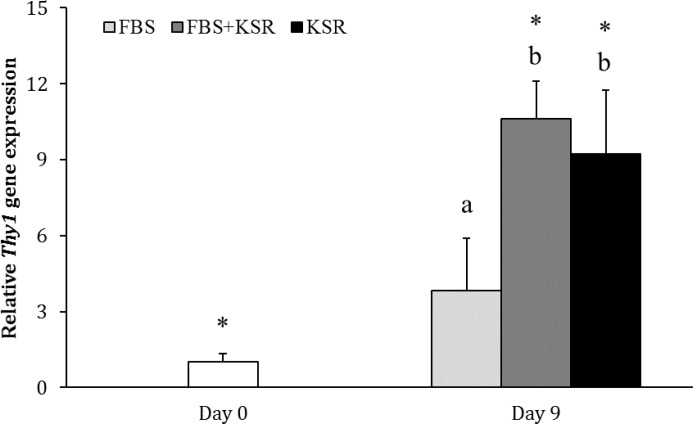
Relative gene expression of *Thy1* in FBS, FBS+KSR and KSR groups (n = 3) on days 0 and 9. Asterisks indicate significant difference within groups between days 0 and 9 (*p *< 0.05). Different letters indicate significant difference between two experimental groups on day 9 (*p *< 0.05

The expression of *Thy1* was not different between days 0 and 9 in FBS group (*p *> 0.05), but it was higher on day 9 than day 0 in FBS+KSR (10.62 ± 1.47 fold; *p* < 0.05) and KSR (9.22 ± 2.52 fold; *p* < 0.05) groups. The gene expression of *Thy1* on day 9 was greater in FBS+KSR and KSR groups as compared with FBS group (*p *< 0.05), but it was not different between FBS+KSR and KSR groups (*p *> 0.05; Fig. 3).  

**Table 3:** Number of colonies in fetal bovine serum (FBS), FBS+ knock-out serum replacement (KSR) and KSR groups on days 3, 6, 9 and 12 of culture. Data are presented as mean ± SD

**Group**	**Days of culture**			
	**3**	**6**	**9**	**12**
**FBS**	1.0 ± 0.7 A	11.4 ± 3.4 A	23.6 ± 6.7 A	26.6 ± 8.6 A
**FBS+KSR**	82.0 ± 11.0 aB	224.8 ± 24.7 bB	438.8 ± 44.9 cB	391.0 ± 25.8 dB
**KSR**	63.0 ± 11.6 aB	159.8 ± 27.4 bC	349.6 ± 28.3 cC	308.0 ± 20.7 cC

abcd different lowercase superscripts within rows indicate significant differences (*p* < 0.05).

AB different uppercase superscripts within columns indicate significant differences (*p* < 0.05).

## Discussion

 Enhancement of SSCs proliferation and self-renewal during *in vitro* culture could help enrich the cell suspension with SSC prior to transplantation, thereby augmenting the efficiency of SSC transplantation.^1^ The experiment 1 showed that replacement of FBS with KSR increased the number of colonies as well as the gene expression of *Thy1*, which has been reported as a conserved marker for undifferentiated spermatogonia including SSCs in a broad series of mammals^16^^-^^20^ and is considered as the best marker for enrichment of SSCs prior to transplantation.^21^ This phenomenon implicates that KSR could not only increase SSCs proliferation but also enhance their self-renewal. Likewise, Garcia-Gonzalo and Izpisúa Belmonte reported that inclusion of KSR in the culture media would favor the expansion and self-renewal of human embryonic stem cells.^13^ Knock-out serum replacement has also been reported to stimulate the proliferation of murine SSCs.^11^

Replacement of FBS with KSR after 3-day culture led to significant increase in the number of colonies. Conversely, replacement of KSR with FBS after a 3-day culture culminated in a decrease in number of colonies over the course of culture. These phenomena implicate that the inferior proliferative effect of FBS as well as the superior proliferative impact of KSR on SSCs are not permanent and irreversible. Nevertheless, experiment 3 revealed that FBS does not contain inhibitory factors diminishing the expansion of SSCs, and its combination with KSR could even synergistically improve SSCs colonization. Perhaps, FBS simply lacks factors essential for proliferation of SSCs. Evaluating the effect of KSR versus FBS for testes organ culture, Sato *et al*. observed progress of spermatogenesis in response to KSR, but not in response to FBS.^12^ Further, it was revealed that this phenomenon was not caused by inhibitory components within FBS; alternatively, it was believed to result from the fact that FBS lacked factors essential for spermatogenesis progression.^12^ Sato *et al*. found lipid-rich albumin as the potential ingredient of KSR, which was essential for progression of spermatogenesis.^12^ Garcia-Gonzalo and Izpisúa Belmonte has also demonstrated lipid-rich albumin as the component responsible for the positive effect of KSR of human embryonic stem cells.^13^ Moreover, it was revealed that the lipids are determining factors, but not albumin in this regard because this simulative effect on self-renewal was trypsin-resistant.^13^ Additionally, lipid-poor albumin did not favor the self-renewal of human embryonic stem cells as did lipid-rich albumin.^13^ Hence, the beneficial impact of KSR on SSCs proliferation in the present study might have resulted from its lipid-rich albumin constituent, which yet requires further studies to be comprehensively addressed.

Cells cultured with KSR or FBS+KSR mostly experienced reduction in colonization after day 9. When the diploid cells become confluent, their mitotic rate would markedly decrease as a result of cell contact, which is referred as contact inhibition.^22^^-^^24^ At this stage, the cells would be insensitive to nutrient renewal.^24^ Accordingly, the decrease in colonization of cells cultured with KSR and FBS+KSR might have caused by confluence and the consequent contact inhibition.

In conclusion, the present study demonstrated that inclusion of KSR in culture medium would markedly increase SSCs proliferation. Further studies are warranted to investigate whether the effect of KSR during *in vitro* culture could lead to improvement in the efficiency of SSCs transplantation. In addition, the results showed that FBS lacked factors essential for proliferation of SSCs.
